# Allopurinol partially prevents disuse muscle atrophy in mice and humans

**DOI:** 10.1038/s41598-018-21552-1

**Published:** 2018-02-23

**Authors:** Beatriz Ferrando, Mari Carmen Gomez-Cabrera, Andrea Salvador-Pascual, Carlos Puchades, Frederic Derbré, Arlette Gratas-Delamarche, Ludovic Laparre, Gloria Olaso-Gonzalez, Miguel Cerda, Enrique Viosca, Ana Alabajos, Vicente Sebastiá, Angel Alberich-Bayarri, Fabio García-Castro, Jose Viña

**Affiliations:** 1 Danish Center for Molecular Gerontology and Danish Aging Research Center, Department of Molecular Biology and Genetics, Aarhus University, Aarhus, Denmark; 2 Freshage Research Group, Department of Physiology. Faculty of Medicine, University of Valencia and CIBERFES, Fundación Investigación Hospital Clínico Universitario/INCLIVA, Valencia, Spain; 3 Servicio de Oncología Médica, Hospital La Fe, Valencia, Spain; 4 Laboratory of Movement Sport and Health Sciences (M2S), University Rennes 2-ENS, Rennes, France; 5 Department of Pathology, University of Valencia, Valencia, Spain; 6 Servicio de Medicina Física y Rehabilitación, Hospital La Fe, Valencia, Spain; 7 Clinica Ypsilon de medicina física y rehabilitación, Valencia, Spain; 8 GIBI 230 (Biomedical Imaging Research Group), La Fe Health Research Institute, Valencia, Spain; 9 QUIBIM SL, Valencia, Spain

## Abstract

Disuse muscle wasting will likely affect everyone in his or her lifetime in response to pathologies such as joint immobilization, inactivity or bed rest. There are no good therapies to treat it. We previously found that allopurinol, a drug widely used to treat gout, protects muscle damage after exhaustive exercise and results in functional gains in old individuals. Thus, we decided to test its effect in the prevention of soleus muscle atrophy after two weeks of hindlimb unloading in mice, and lower leg immobilization following ankle sprain in humans (EudraCT: 2011-003541-17). Our results show that allopurinol partially protects against muscle atrophy in both mice and humans. The protective effect of allopurinol is similar to that of resistance exercise which is the best-known way to prevent muscle mass loss in disuse human models. We report that allopurinol protects against the loss of muscle mass by inhibiting the expression of ubiquitin ligases. Our results suggest that the ubiquitin-proteasome pathway is an appropriate therapeutic target to inhibit muscle wasting and emphasizes the role of allopurinol as a non-hormonal intervention to treat disuse muscle atrophy.

## Introduction

Muscle atrophy occurs when protein degradation rates exceed protein synthesis and may take place in adult skeletal muscle in a variety of conditions, including denervation, cancer, sepsis, heart failure, aging, bed rest, immobilization, and inactivity^1^. Research into muscle atrophy is of high clinical relevance because the treatment of many diseases involves a reduction in physical activity and in some cases restriction of movement of patients^2^.

Disuse promotes atrophy of skeletal muscle by stimulating protein breakdown^3^. This process involves the activation of the ubiquitin-proteasome pathway^2^. This pathway includes two critical muscle-specific ubiquitin ligases: muscle RING finger 1 (MuRF-1) and muscle atrophy F-box (MAFbx)^4,5^. They regulate the degradation of skeletal muscle proteins such as calcineurin, myoD, troponin-I, titin, and myosin heavy and light chains^6,7^. The ubiquitin-proteasome system is required to remove sarcomeric proteins due to changes in muscle activity. It is constitutively operative in normal skeletal muscle and is responsible for the turnover of most soluble and myofibrillar muscle proteins^8^. The activity of this pathway is markedly increased in atrophying muscle due to transcriptional activation of ubiquitin, of several proteasomal subunit genes, and of MAFbx and MuRF-1^4^. Importantly, the rate of muscle atrophy is markedly reduced by targeted inactivation of these gene products^9^. Another important ubiquitin ligase involved in skeletal muscle atrophy is Casitas B-lineage lymphoma b (Cbl-b)^10,11^. Upon induction, Cbl-b interacts with, and degrades, the IGF-1 signaling intermediate insulin receptor substrate-1 (IRS-1). IRS-1 is a docking protein for several signaling intermediates including p85, the regulatory subunit of phosphatidylinositol 3-kinase (PI-3K). PI-3K activation leads to phospholipid generation in the plasma membrane, which recruits and activates protein kinase B (Akt), leading to activation of mammalian target-of rapamycin (mTOR) and ribosomal protein S6 Kinase (p70S6K) that results in an increase in protein translation initiation and ribosome biogenesis^12^. The mechanism of muscle atrophy mediated by Cbl-b does not appear to involve the degradation of muscle component proteins, but rather to impair muscle protein synthesis by an increase in degradation of signaling molecules^13^.

Two major redox signaling pathways control the activation of muscle ubiquitin ligases. One is mediated by class O type of forkhead transcription factors (FoxO) that upregulate MuRF-1 and MAFbx. FoxO3 is phosphorylated and inactivated by Akt^14^. It has been shown that several autophagy-related genes (LC3-II, Beclin-1, and p62) are downstream FoxOs^15^. The second pathway involves the nuclear factor-κB (NF-κB), which is known to mediate the inflammatory response and which in turn is able to induce the activation of MuRF-1^16^. However, the specific contribution of oxidative stress to muscle atrophy and wasting is not completely clear^17,18^. It has been shown that the ubiquitin-proteasome system is upregulated by reactive oxygen species and inflammation^19^. However, regarding the impact of antioxidant treatments on muscle atrophy, contradictory results have been obtained^17,18^. Allopurinol is an inhibitor of the free radical generating enzyme xanthine oxidoreductase (XOR). It is used in clinical practice to treat hyperuricemia^20^. We have previously reported that allopurinol prevents muscle damage during exhaustive physical exercise^21–23^ and during hindlimb unloading by inhibiting the p38MAPK-MAFbx pathway^24^.

The major aim of our study was to determine the role of allopurinol in the prevention of muscle atrophy induced by two weeks of immobilization, in both mice and humans, and the molecular mechanism involved in this prevention.

## Results

### Allopurinol prevents unloading muscle atrophy in mice

**Figure 1:**
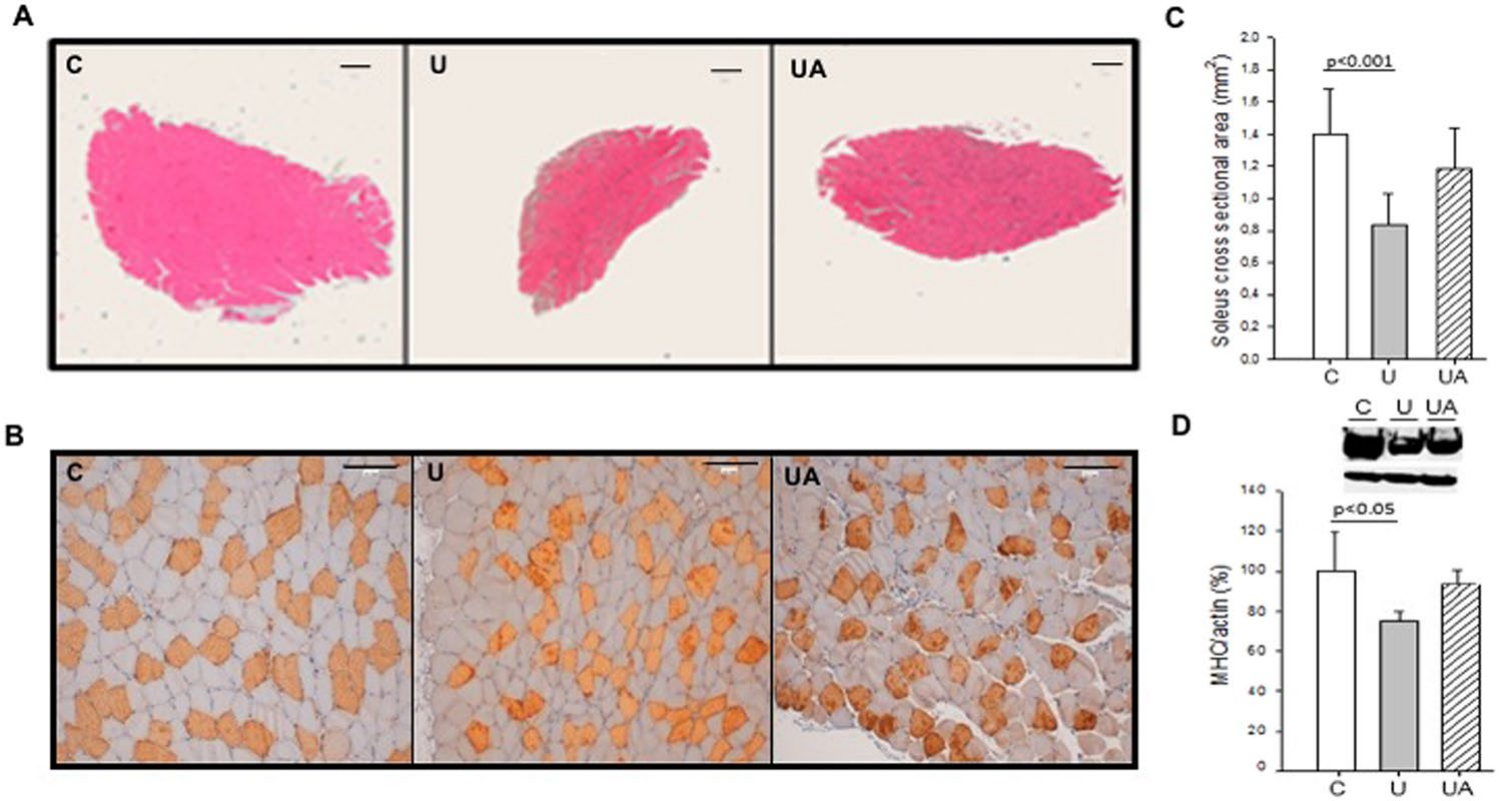
Allopurinol-treated mice are partially protected against hindlimb unloading-induced skeletal muscle atrophy. (**A**) Hematoxylin and eosin staining (Original magnification, 4×. Scale bar, 100.00 µm). (**B**) Myosin staining specific of MHC (type I fibers were deeply stained, while type II were lightly stained. Original magnification 20×. Scale bar, 100.00 µm). (**C**) Shows quantitative analysis of the cross-sectional area of the soleus muscle. (**D**) Representative western blotting and densitometric analysis of soleus MHC I (full-length blots are included in supplementary information. See Supplementary Figure 1). Actin was used as a loading control. Data are shown as mean ± SD. C = Control (n = 5–7); U = Unloading (n = 5–7); UA = Unloading treated with allopurinol (n = 5–7).

In our study 14 days of hindlimb unloading in mice induced a small (not significant) decrease of gastrocnemius muscle/body weight ratio (mg/100 g): control group (516.7 ± 79.3), unloaded group (447.3 ± 50.0), and unladed group treated with allopurinol (511. 3 ± 31.5). Antigravity muscles are most affected by immobilization. The greatest reduction in the cross-sectional area (CSA) of the lower limb muscle groups during disuse protocols has been found in the soleus muscle^25–28^. Figure 1 shows that unloading causes a significant decrease in the CSA (~41%, p < 0.001) and minimum transverse diameter in type I fibers (~25%, p < 0.001) of the mice soleus muscle (Fig. 1, panels A–C). We further tested the protein content of the slow Myosin Heavy Chain (MHC) protein, an important component of the sarcomere. Figure 1, panel D, shows that after unloading, there is a significant decrease in the protein content of MHC I in soleus muscle. Allopurinol treatment partially prevents the decrease in both, cross sectional area (~14%) and MHC I protein content (~19%) in the muscle. However, no protection was found in the minimum transverse diameter in all the fibers and specifically in type I fibers (data not shown). The content of α-actin was not altered in any experimental group and it was used as a loading control^29^. The effectiveness of the treatment with allopurinol was determined by measuring XO activity in plasma: it rose significantly from 1.0 ± 0.1 U·L^−1^ in the control group to 3.4 ± 1.7 U·L^−1^ in the unloaded animals (p < 0.001). As expected, the activity of the enzyme was barely detectable in the unloaded animals treated with allopurinol 0.1 ± 0.2 U·L^−1^ (p < 0.001).

### Allopurinol modulates inflammation and oxidative stress during hindlimb unloading

**Figure 2:**
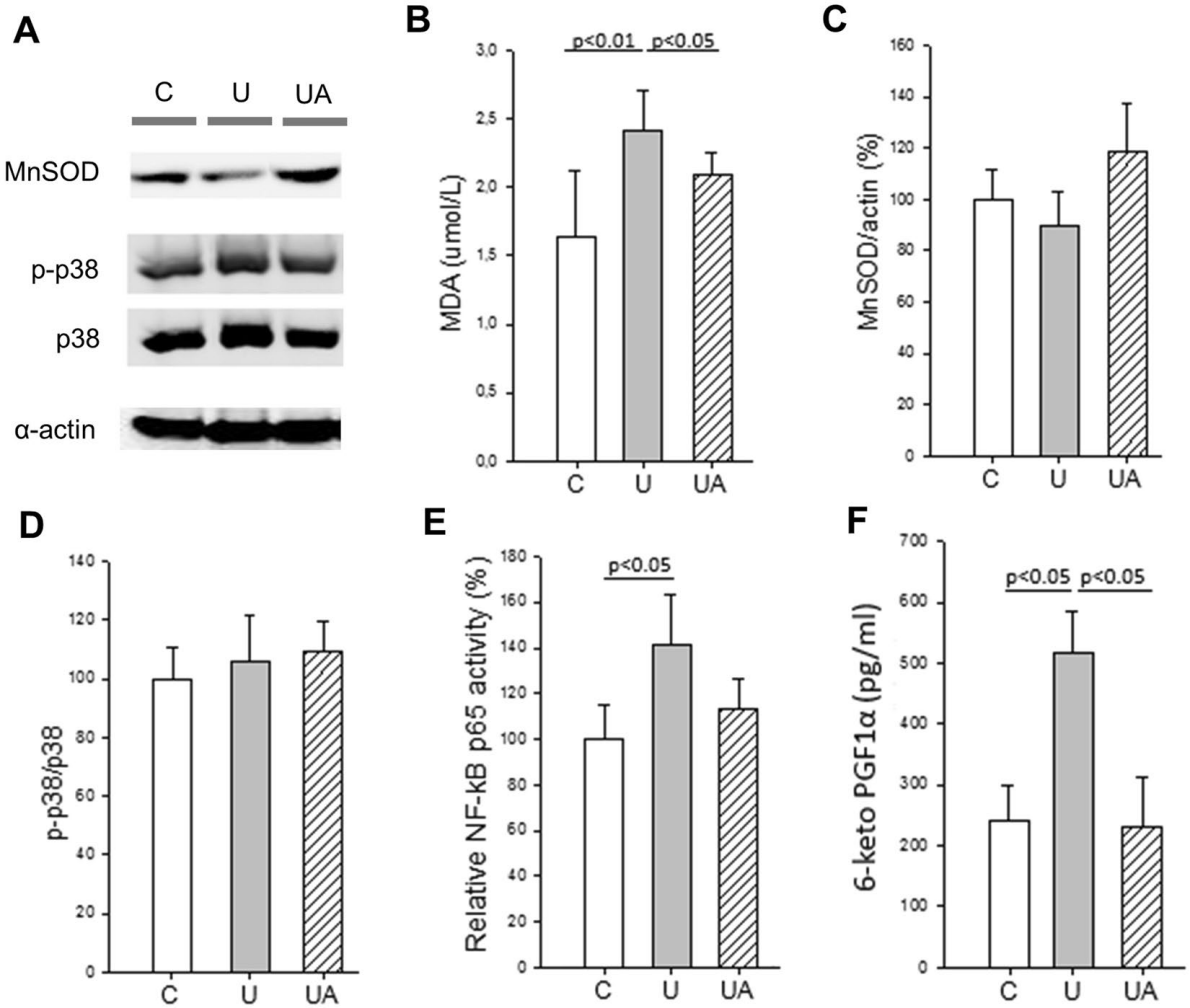
Allopurinol-treated mice are partially protected against hindlimb unloading-induced inflammation and oxidative stress. (**A**) Representative western blotting of soleus proteins (full-length blots are included in supplementary information. See Supplementary Figure 2). (**B**) Shows plasma MDA levels measured by HPLC. (**C** and **D**) show the densitometric analysis quantified by using relative expression in arbitrary units of MnSOD (**C**), and p-p38 (**D**). (**E** and **F**) Show NF-κB p65 binding activity (**E**), and plasma 6-keto prostaglandin F1α (**F**) Determined by ELISA. Actin was used as a loading control. Data are shown as mean ± SD. C (n = 4–7), U (n = 4–6), and UA (n = 4–6).

We next evaluated whether the protective effect of allopurinol in unloading atrophy was due to the modulation of oxidative stress and inflammation. To assess oxidative damage, we determined malondialdehyde (MDA) as a marker of lipid peroxidation in plasma. Hindlimb unloading caused a significant increase in the plasma lipid peroxidation that was prevented in the allopurinol treated animals (Fig. 2, panel B). To test the muscle adaptation to oxidative stress we also determined the protein levels of the antioxidant enzyme MnSOD. Hindlimb unloading caused minor changes in the protein levels of MnSOD in soleus muscle (Fig. 2, panels A and C). Contrary to our previous results in rats^24^, unloading did not cause an activation of the redox sensitive p38 MAPKs pathway in the skeletal muscle (Fig. 2, panels A and D). Reactive oxygen species (ROS) are thought to induce muscle atrophy through the activation of the pro-inflammatory transcription factor NF-κB. We measured the DNA binding activity of the p65 subunit of the transcription factor and a significant increase (p < 0.05) was detected in the gastrocnemius muscle in the unloaded group. Allopurinol treatment prevented this activation of NF-κB. No differences were found in the p65 binding activity between the control group and the unloaded animals treated with allopurinol (Fig. 2, panel E).

NF-κB regulates the expression of cyclooxygenase-2 (COX-2) mRNA^30^. Prostaglandins (PGs), synthesized as a result of the enzymatic activity of COX, function as classic mediators of inflammation^31^. Thus, we tested whether hindlimb unloading induced an increase in the plasma concentration of the 6-keto prostaglandin F1α. Figure 2 shows that it was significantly elevated in the unloaded mice and that treatment with allopurinol completely prevented this increase (Fig. 2, panel F). The content of α-actin was not altered in any experimental group (Fig. 2, panel A).

All these results indicate that hindlimb unloading results in an increase in oxidative stress and inflammation and that allopurinol prevents it.

### Allopurinol up-regulates IGF-1/Akt and subsequently down-regulates muscle atrophy-related E3 ubiquitin ligases

**Figure 3:**
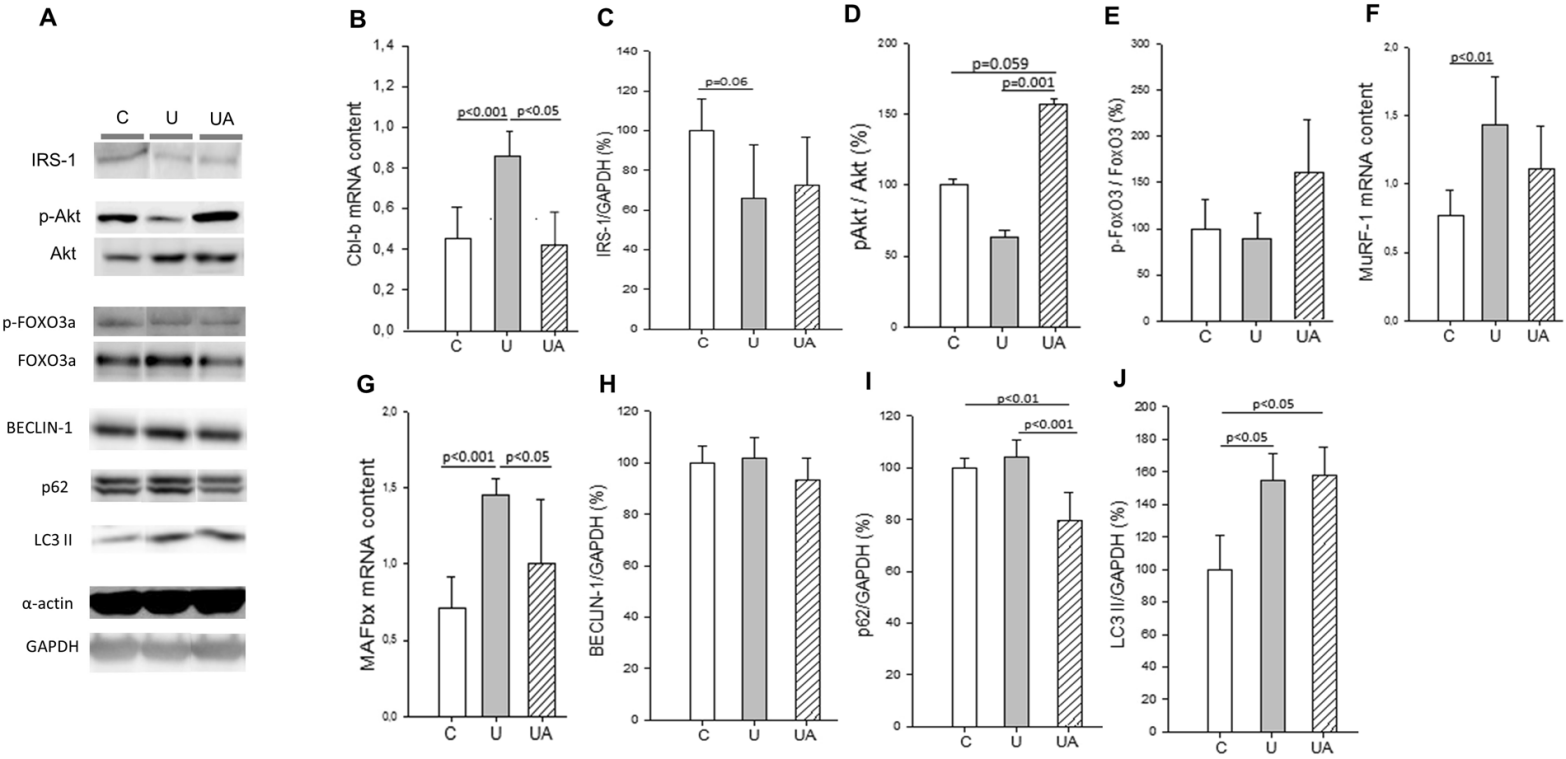
Allopurinol up-regulates the IGF-1/Akt pathway and down-regulates muscle atrophy-related E3 ubiquitin ligases in skeletal muscle. (**A**) Representative western blotting of soleus proteins (full-length blots are included in supplementary information. See Supplementary Figure 3). (**B**) Shows mRNA levels of Cbl-b in soleus muscle. (**C** to **E**) Show the densitometric analysis quantified using relative expression in arbitrary units of IRS-1 (**C**), p-Akt (**D**), p-FoxO3 (**E**). (**F** and **G**) Show the soleus muscle mRNA levels of MuRF-1 (**F**) and MAFbx (**G**). (**H** to **J**) Show the densitometric analysis quantified using relative expression in arbitrary units of Beclin-1 (**H**), p62 (**I**), and LC3-II (**J**). Actin and GAPDH were used as a loading control in the western blots, as indicated. Data are shown as mean ± SD, C (n = 4–6), U (n = 4–5), and UA (n = 4–5).

We next evaluated whether the protective effect of allopurinol on the prevention of unloading muscle atrophy was due to the modulation of the IGF-1/Akt signaling cascade. This pathway is known to play a role in regulating muscle mass^32^ and is decreased in muscle atrophy induced by cancer cachexia^33^ and hindlimb unloading in young^34^ and old animals^29^. Cbl-b is a ubiquitin ligase involved in skeletal muscle atrophy^11^. Upon induction, it degrades IRS-1 leading to the inactivation of Akt. We found a significant increase in Cbl-b (mRNA levels) in the soleus muscle of the unloaded animals that was completely prevented in the allopurinol treated ones (Fig. 3, panel B). We determined the protein levels of IRS-1 (Fig. 3, panel A and C) but no differences were found between the groups. As expected, Akt was down-regulated in the unloaded mice. By contrast, we observed a very significant increase (p = 0.001) in the phosphorylation of Akt in the allopurinol-treated animals even when compared to the controls (Fig. 3, panels A and D). Akt can promote muscle growth^35^ and simultaneously block protein degradation^36^ via transcription factors of the FoxO family^12^. Akt phosphorylates FoxO proteins, promoting their export from the nucleus to the cytoplasm which leads to the inhibition of FoxO-dependent gene expression. Thus, we measured the phosphorylation of FoxO3 in whole muscle extracts of soleus muscle. We did not find any change in the p-FoxO3/FoxO3 ratio in the unloaded group and those treated with allopurinol (Fig. 3, panels A and E). The protein levels of the autophagy-related gene LC3-II was significantly increase in the unloading group, this induction was not prevented by the treatment with allopurinol (Fig. 3, panel A and J). However, we did no find a clear induction of Beclin-1, and p62 (panels A, H and I) in our model. We finally determined the mRNA levels of the two atrophy-related E3 ubiquitin ligases, MuRF-1 and MAFbx, and as shown in Fig. 3 (panels F and G) we found a significant increase in the gene expression of both enzymes in the soleus muscle of the unloaded animals. In the case of MuRF-1 (panel F) no differences were found in the mRNA levels between the control animals and those unloaded and treated with allopurinol, but in the case of MAFbx (panel G) allopurinol led to a significant decrease (p < 0.05) in the unloading-induced expression of the enzyme.

The content of α-actin or GAPDH was not altered in any group (Fig. 3, panel A). These results indicate that the loss of muscle mass in our model occurs primarily through enhanced protein breakdown due to the activation of the ubiquitin-proteasome pathway. To sum up, allopurinol protects against loss of muscle mass by inhibiting the expression of MuRF-1 and MAFbx through a mechanism that involves Cbl-b, Akt and NF-κB.

Some of the previous results published by our research group, using rats as a model^24^, were confirmed in this new study using mice. Based on that, we designed a clinical trial to study muscle atrophy in ankle sprain patients during immobilization.

### Allopurinol prevents muscle atrophy in ankle sprain (grade II) patients during immobilization. Results of a clinical trial: EudraCT 2011-003541-17

We performed a prospective, interventional, randomized study of the muscle atrophy determined by MRI during lower leg immobilization following ankle sprain (grade II) in young (aged between 25–40 years) male subjects. Following randomization, 13 patients were treated with allopurinol and 12 did not receive any treatment (control) during all the immobilization period (See subjects flow diagram in Supplementary Figure 4). We first determined uric acid levels in all patients to test the effectiveness of the allopurinol treatment. We did not find any difference between the uric acid levels in the control group before 5.9 ± 0.8 mg·dL^−1^ and after the immobilization 5.9 ± 0.7 mg·dL^−1^. As expected, a significant decrease was found in allopurinol-treated subjects 3.7 ± 0.5 mg·dL^−1^ after immobilization when compared with their values before it 5.7 ± 0.6 mg·dL^−1^ (p < 0.05).

**Figure 4:**
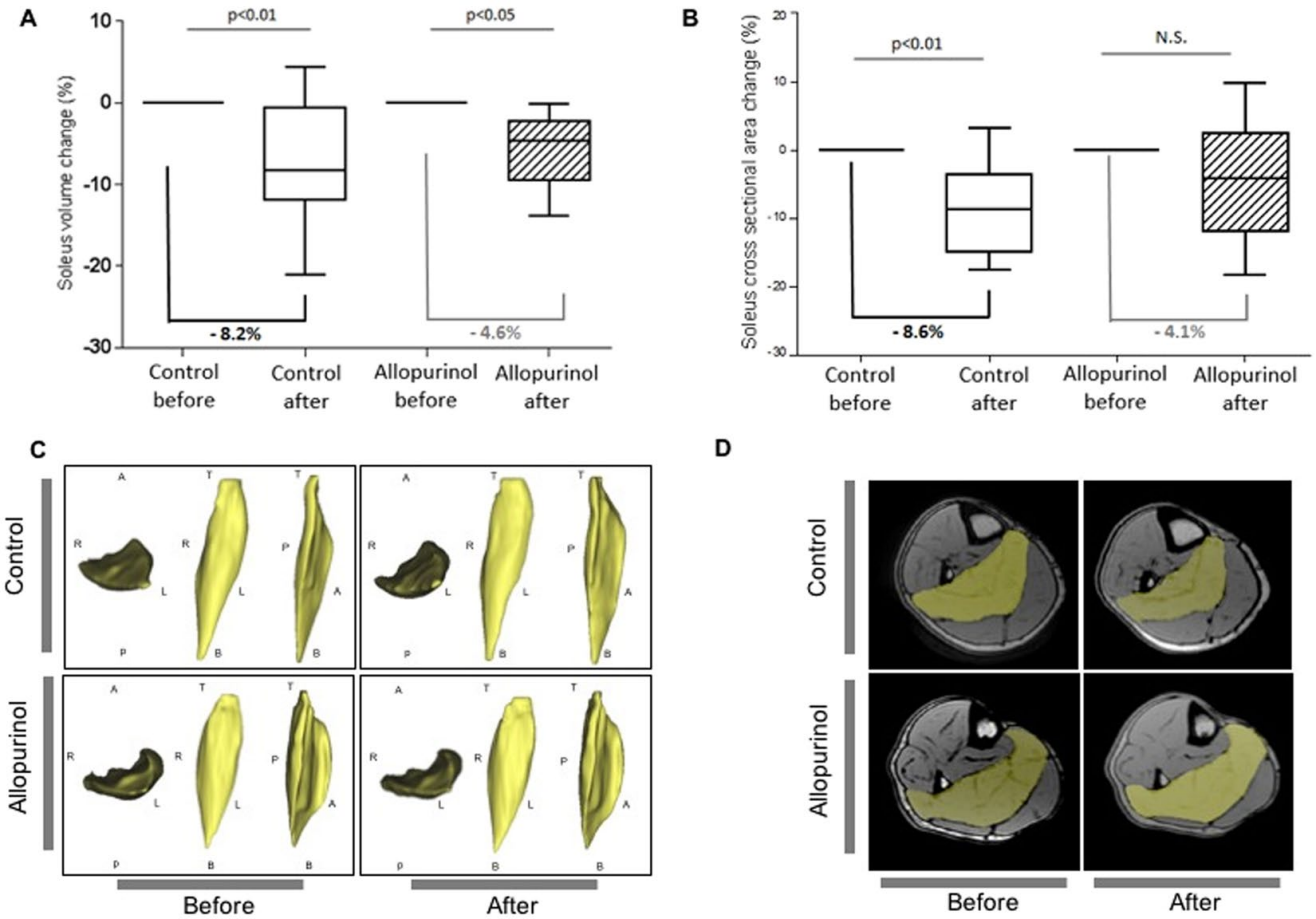
Allopurinol-treated ankle sprain patients are partially protected against unloading-induced skeletal muscle atrophy. (**A)** Quantification of the changes in soleus muscle volume during lower leg immobilization with a posterior ankle splint. **(B)** Quantification of the changes in soleus muscle CSA during lower leg immobilization with a posterior ankle splint. **(C)** Representative volume images after analysis and segmentation of the soleus muscle in control and allopurinol-treated subjects. R = right, L = left, A = anterior, P = posterior, T = top, B = bottom. **(D**) Representative magnetic resonance images of control and allopurinol-treated subjects. The soleus muscle is shown in yellow.

Figure 4 shows that 15 days of unilateral lower limb unloading causes a significant decrease in the volume (8.2%, p < 0.01) (Fig. 4, panels A and C) and cross sectional area (8.6%, p < 0.01) of the soleus muscle (Fig. 4, panels B and D). Treatment with allopurinol partially prevented the decrease in the CSA of the soleus muscle in the immobilized patients (4.1%) making the difference between before and after immobilization non-significant (See Fig. 4B). No prevention was found in the soleus volume (4.6%) (See Fig. 4A).

We did not find any significant differences between the control and allopurinol group in any inflammatory, liver or muscle damage marker. Results related to blood parameters analyzed in the clinical trial are available in Supplementary Table 1.

## Discussion

Skeletal muscle atrophy can occur in response to immobilization, in pathological conditions, and in normal aging (primary sarcopenia)^37^. In human studies, quantification of muscle CSA has been commonly used in assessment of muscle atrophy. Previous data indicate that allopurinol reduces oxidative stress^23^, prevents muscle damage^38^, and is associated with a greater degree of improvement in function as measured by the Barthel score during rehabilitation in an older inpatient population^39^. We have found that the soleus muscle CSA after 15 days of immobilization with a posterior ankle splint decreased significantly (8.6%, p < 0.05) in the control group of patients. Treatment with allopurinol prevented partially this decrement in the immobilized patients (4.1%) (See Fig. 4). To highlight the clinical significance of the allopurinol effect, we compared it with exercise, the best known way to prevent muscle mass loss in disuse human models^40^. Table 1 shows the main human studies in which the plantar flexors (or specifically soleus muscle) atrophy induced by disuse have been treated with resistance exercise. The decline in the muscle size of our patients parallels data from a number of studies that report decreases from 7 to 15% after 20 days of bed rest or unilateral lower limb unloading. Table 1 shows that our intervention (300 mg of allopurinol daily), seems to be as effective as exercise for atrophy prevention^25–27,41,42^. In this regard, we would like to highlight that the atrophic response seems to be higher in the soleus muscle than in the medial and lateral gastrocnemius muscles, in both the animal and human models^26^. Thus, preventing atrophy is especially challenging in the soleus muscle and was the major aim of our study.

**Table 1:** Summary of the human studies in which the plantar flexor or the soleus muscle atrophy has been treated with exercise. Comparison with the results achieved in our study.

	N° of subjects	Muscle	Protocol	Treatment	Control			Intervention		
					Pre (cm^2^)	Post (cm^2^)	% Change	Pre (cm^2^)	Post (cm^2^)	% Change
Our study	25 ankle sprain patients	Soleus CSA	15 d of unilateral lower limb immobilization	300 mg/day of allopurinol	31.2 ± 4.6	28.6 ± 5.8	8.6 (p < 0.05)	28.8 ± 4.6	27.7 ± 5.8	4.1 (NS)
^26^	9 healthy men	Soleus PCSA	20 d of bed rest	Isometric leg-press	41.2 ± 9.5	35.0 ± 6.1	15.0 (p < 0.05)	45.1 ± 9.2	40.3 ± 5.1	10.6 (p < 0.05)
^25^	15 healthy men	Soleus PCSA	20 d of bed rest	Dynamic leg press: knee extension and plantar flexion	40.3 ± 7.4	35.4 ± 5.2	12.1 (p < 0.01)	56.9 ± 18.1	51.3 ± 17.6	9.8 (p < 0.01)
^41^	16 healthy men	Plantar flexor CSA	21 d of unilateral lower-limb suspension	High-intensity resistance-training	—	—	7.0 (p < 0.05)	—	—	~0%
^27^	12 healthy men	Plantar flexor PCSA	20 d of bed rest	Leg press and plantar flexion resistance training	138.3 ± 19.0	122.2 ± 24.3	12.7 (p < 0.05)	117.9 ± 8.0	115.0 ± 10.5	3.2 (NS)
^42^	11 healthy men	Plantar Flexor CSA	20 d of unilateral lower limb suspension	Cycling training (80% of VO_2peak_)	—	—	11.2 (p < 0.05)	—	—	7.0 (p < 0.05)

PCSA: Physiological Cross Sectional Area. PCSA = Muscle Volume × Cosine of the muscle fiber pennation angle × (fibre length)^−1^.

NS: Not significant.

We could not obtain soleus muscle biopsies from the patients. Comparison of disuse atrophy in rodents and humans suggests that there are considerable similarities, and that the biggest difference appears to be in the rate at which atrophy occurs, with loss of mass in rodents being considerably faster than in humans^2^. Thus, to examine the molecular pathways modulated by allopurinol in skeletal muscle we performed studies in mice.

We found that allopurinol partially prevented the decrease in the soleus CSA after 14 days of hindlimb unloading in mice. This prevention was approximately 14%. However, no effect of the treatment was found on the loss of the minimum transverse diameter in soleus muscle type I fibers. This result suggests that actual loss of muscle type I fibers, rather than simple atrophy, may be responsible for the differences in the CSA. In fact, we found a partial prevention in the loss of MHC I protein content in soleus muscle in the unloaded animals treated with allopurinol (See Fig. 1). There are evidences showing the protective role of allopurinol in the prevention of muscle dysfunction caused by prolonged unloading^43^ or mechanical ventilation^44^. Moreover, it has previously been found that inhibition of xanthine oxidase with allopurinol reduces wasting and improves survival in a rat model of cancer cachexia^45^.

Under atrophy conditions there is a shift in the balance of protein synthesis and degradation resulting in a net loss of muscle proteins^2^. There is strong evidence in rodent models^46–48^ and in humans^49,50^ of disuse showing that the rate of basal protein synthesis declines immediately after unloading and stays at a suppressed level for the duration of the disuse. The cellular mechanisms responsible for regulation of protein synthesis in adult skeletal muscle include the activation of the IGF-1/Akt signaling cascade^9^.

Here we report a significant increase in the mRNA levels of Cbl-b in the soleus muscle of the unloaded animals that was completely prevented by allopurinol. Cbl-b-deficient mice are resistant to unloading-induced atrophy^11^ and its inhibition with delphinidin (an anthocyanin), suppresses the atrophy of skeletal muscle in a rodent model of disuse^51^. IRS-1 is the docking protein for the regulatory subunit of PI-3K, the kinase that activates Akt. Reduced daily ambulatory activity decreases insulin-stimulated Akt phosphorylation, peripheral insulin sensitivity and leg lean mass^52^. No changes were found in the IRS-1 protein levels but, as expected, Akt was down-regulated in our model of unloading. By contrast, we observed a very significant increase in the phosphorylation of Akt in the allopurinol-treated animals even when compared to the controls. This effect of allopurinol on Akt has been reported in other models^53^. Moreover, it has been found that treatment with febuxostat (a novel non-purine selective XO inhibitor) attenuates cachexia progression in tumor-bearing rats through the increase in the pAkt/Akt ratio in gastrocnemius muscle^33^. Akt can promote muscle growth and simultaneously block protein degradation via phosphorylation of FoxO proteins that regulate the expression of atrophy-related genes^12,36^. Thus, we measured the phosphorylation of FoxO3 in whole muscle extracts of soleus muscle. We did not find changes in the p-FoxO3/FoxO3 ratio in any experimental group. We also determined the mRNA levels of the two atrophy-related E3 ubiquitin ligases, MuRF-1 and MAFbx, and as shown in Fig. 3, we found a significant increase in the gene expression of both enzymes in the soleus muscle of the unloaded animals that was prevented in the allopurinol treated ones. The protective effect of allopurinol on the induction of MuRF-1 and MAFbx has previously been found in two different animal models of disuse: cachexia^45^ and hindlimb unloading^24^. Apart from the ubiquitin-proteasome, the autophagy-lysosome is another important system involved in protein degradation during muscle atrophy^54^. We only found an increase in the protein levels of LC3-II in the skeletal muscle of the unloaded animals that was not prevented with allopurinol.

Our results indicate that allopurinol protects against the loss of muscle mass by the inhibition of MuRF-1 and MAFbx through a mechanism that involves Cbl-b, and Akt.

A pivotal role has been ascribed to oxidative stress in determining the imbalance between protein synthesis and degradation in muscle atrophy^17,18^. We found a significant increase in one marker of oxidative damage in plasma (MDA) and an activation of the redox sensitive transcription factor NF-κB in the muscle of the unloaded animals that was prevented by allopurinol. However, we did not find changes in the phosphorylation of the redox sensitive MAPKinase p38 in the soleus muscle of the unloaded animals. This negative finding could be due to an earlier time point activation of the signals.

Classically, it has been considered that ROS induce proteasome expression through activation of NF-κB. Both antioxidant (such as MnSOD) and inflammatory genes (such as COX-2) require NF-κB binding to activate their transcription^55^. We did not find an increase in the protein levels of MnSOD in the soleus muscle in the unloaded group, however we did find a very significant increase in the plasma concentration of the 6-keto prostaglandin F1α, which is synthesized as a result of the enzymatic activity of COX. Using KO models, it has been demonstrated that XOR activity can regulate the level of COX-2^56^. Indeed, previous evidence supports a redox-dependent induction of COX-2^57,58^.

Our results indicate an activation of the NF-κB transcription factor accompanied by an increase in the levels of the inflammatory mediator 6-keto prostaglandin F1α during unloading that is prevented by allopurinol. Based on the low extent of oxidative damage detected in our model, we consider that the protective effect of allopurinol may be mediated by the modulation of ROS-dependent (XOR inhibition) but also ROS-independent intracellular pathways.

**Figure 5:**
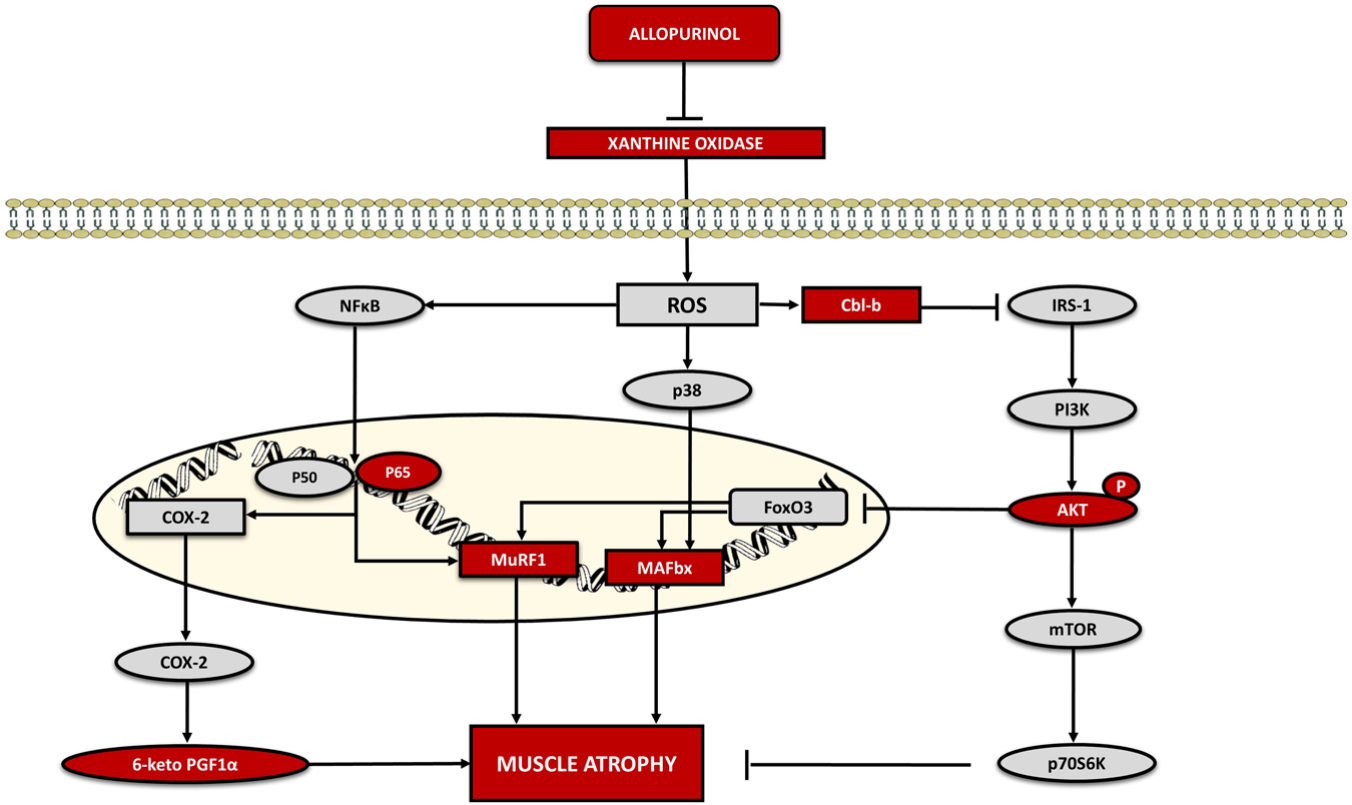
Proposed signaling pathways activated during muscle atrophy and the effect of allopurinol administration.

Figure 5 shows a schematic representation highlighting the main signaling pathways activated during muscle atrophy in our study and the effect of allopurinol administration.

## Conclusion

Disuse muscle atrophy is a common clinical problem. It will likely affect every person in his or her lifetime and currently there are no good therapies to treat it. Only resistance exercise can be used to promote recovery of mass/strength following disuse atrophy. Our results show that allopurinol treatment partially prevents soleus muscle atrophy after two weeks of unloading in both mice and humans. Allopurinol protects against the loss of muscle mass by the inhibition of MuRF-1 and MAFbx through a mechanism that involves Cbl-b, Akt and NF-κB. Thus, allopurinol may become useful to prevent muscle atrophy in unloading models, such as bed rest, limb immobilization/suspension, and even reduced physical activity.

## Limitations of the Study

Due to technical reasons, we could not perform dynamometry testing in either the animal or the human study. It would have been interesting to know whether the prevention in the muscle loss by treatment with allopurinol was accompanied by prevention in muscle functionality, which is a very relevant clinical parameter. We could not obtain soleus muscle biopsies from the patients which has prevented us from analyzing the cell signaling pathways involved in atrophy in the human study. Although the primary outcome of muscle mass was made by blinded observers, the inclusion of a placebo would have eliminated the possibility that subjects knowing they were in the treatment group might have mitigated their atrophy by being more active. Finally, we consider that the number of patients that participated in the clinical trial is noteworthy. However, there was a 28% drop out rate in the trial. Therefore, the human results should be viewed as a Phase 2, preliminary trial.

## Methods

### Studies approval

The animal study was approved by the Research Ethics Committee of the University of Valencia (License reference: A1336337814210). The human study was approved by the Research Ethics Committee of the “Hospital Universitario la Fe de Valencia”. The study was carried out in accordance with the ethical principles in the Declaration of Helsinki. Informed consent was obtained from each participant who signed it after fully understanding the procedures.

### Animals

Twenty-one male C57Bl/6J wild-type mice (~35 g, 3 months old) from Charles River, were randomly assigned to one of three experimental groups: ground control (C) (n = 7); hindlimb unloaded (U) (n = 7); hindlimb unloaded treated with allopurinol (UA) (n = 7). Allopurinol was dissolved in drinking water as previously described^24^.

### Hindlimb Unloading protocol

After a 48 h of pre-treatment with allopurinol, the animals were subjected to the hindlimb unloading protocol using the Morey-Holton and Globus method^59^. Briefly, an orthopedic tape-adhesive surrounding the cleaned and dried tail was attached to the top of the cage through a swivel shackle (enabled the animals a 360° range of motion) and a nylon monofilament line. The mouse’s hindlimbs were elevated and adjusted to prevent it touching the ground of the cage. After 14 days of unloading, the animals were anesthetized while hindlimb-unloaded. Then, blood was obtained by venous puncture into heparin and EDTA tubes. Right soleus and gastrocnemius muscles were dissected, weighed and immediately frozen in liquid nitrogen, and stored at −80 °C. Left soleus muscles were fixed in 4% formalin to perform histological studies, after being weighed.

### Xanthine oxidase activity

Xanthine oxidoreductase activity was measured in plasma as previously described^20,60^.

### Western Blotting

Protein extracts from muscle lysates were separated into SDS-polyacrylamide gel electrophoresis as described elsewhere^61^. Membranes were incubated with the following primary antibodies: anti-Myosin (1:5000, Millipore, MAB1628), anti-MnSOD (1:5000, Assay Designs ADI-SOD-110), anti-p38 (1:1000, Cell Signaling, 9212S), anti-phospho-p38 (1:1000, Cell Signaling, 9211S), anti-Akt (1:1000, Cell Signaling 8596S), anti-phospho-Akt (1:1000, Cell signalling, 9271s), anti-FoxO3 (1:500, Cell Signaling, 12829S), anti-phospho-FoxO3 S (1:250, Millipore 06-953), anti-α-actin (1:700, Sigma Aldrich, A2172), anti-IRS1 (1:500, Millipore, 06-248), anti-Beclin-1 (1:1000, Cell Signaling, 3495S), anti-p62/SQSTM (1:1000, Sigma Aldrich, P0067), anti-LC3-II (1:1000, Cell Signaling, 3868S), anti-GAPDH (1:10000, Sigma Aldrich, G9545). The proteins of interest were normalized to the α-actin or to the GAPDH expression for each densitometry. We have included only a representative Western blot in each figure. Full-length blots are included in supplementary information. Electrophoretic gels and blots were analyzed with Image Gauge or ImageJ softwares, without any manipulation. Only, in case that it was needed, we change slightly the contrast of the image, but always in a linear way to all the samples and never getting a saturated image or deleting any band. For the figures, the blot image was open in Paint software to be cropped and, to assemble the final version of the image.

### Malondialdehyde levels

Lipid peroxidation determination as MDA in plasma was performed as previously described^62^.

### Histology

Cross sectional areas were measured by hematoxylin and eosin staining as previously described^24^. Muscle fiber types were identified by immunohistochemical analysis using anti-myosin heavy chain I antibody (1:4000, Sigma Aldrich). Counterstaining was performed with hematoxylin. The minimum transverse diameter in type I fibers was determined measuring the minimum straight line segment that joins two opposite points of the fiber passing through its center. All the histological sections were visualized using a LEICA DMD 108 light microscope. Image analysis was carried out with the Image Pro.Plus.6 software. Figure 1 (panels A and B) and Fig. 4 (panels C and D) were assembled with Paint software. The images size was adjusted as a whole image (in all the samples) to the paper format (Photographs of 2048 × 1536 pixels).

### NF-κB p65 DNA binding activity

NF-κB was determined in nuclear extracts from gastrocnemius muscle according to a modified protocol by Dignam *et al*.^63^ using an ELISA-based TransAM NF-κB p65 assay kit (Active Motif, Carlsbad, CA, USA) in accordance with the manufacturer’s instructions. Sample absorbance was read at 450 nm on a multiwell microplate reader (Spectra Max plus 284, Molecular Devices). Wild-type and mutated consensus oligonucleotides were used as competitors for NF-κB binding to ensure specificity of the reaction as per the manufacturer’s instructions. All the tests of the samples were run in duplicate, and the average value was used for data analysis.

### Assay of 6-keto-prostaglandin F1α

The concentration of stable hydrolysis product 6-keto-prostaglandin F1a in plasma was assessed in duplicate by a commercial enzyme immunoassay kit (Cayman Chemical) in accordance with the manufacturer’s instructions. Sample absorbance was read at 450 nm on a multiwell microplate reader (Spectra Max plus 284, Molecular Devices). Sample absorbance was directly compared between individual samples on individual plates and normalized to a standard amount of positive control. The production of prostacyclin was expressed as ng prostacyclin/mg protein.

### RNA isolation, reverse transcription and PCR

Total RNA from was extracted as previously detailed^24^. Samples were reverse transcribed and real-time PCR was performed with an ABI 7900 sequence-detection system (Applied Biosystems, Madrid, Spain). Real-time PCR was performed using Maxima™ SYBR green/ROX qPCR Master Mix (Fermentas, Madrid, Spain). A melting curve analysis was performed to confirm that only the specific products were amplified. The threshold cycle was converted to a relative gene expression by using a standard curve.

Primers for amplifying specific fragments of the genes were obtained from Thermo Fisher Scientific GmbH (Ulm, Germany): MAFbx-F, 5′-GCAAACACTGCCACATTCTCTC-3′; MAFbx-R, 5′CTTGAGGGGAAAGTGAGACG-3′; MuRF-1-F,5′-ACCTGCTGGTGGAAAACATC-3′; MuRF-1-R,5′-CTTCGTGTTCCTTGCACATC-3′; Cbl-b-F, 5′-GAGCCTCGCAGGACTATGAC-3′; Cbl-b-R,5′-CTGGCCACTTCCACGTTATT-3′; Cyclophilin-F, 5′-AGCATGTGGTCTTTGGGAAGGTG-3′; Cyclophilin-R, 5′-CTTCTTGCTGGTCTTGCCATTCC-3′.

For each sample, the expression of the target gene was normalized with the cyclophilin mRNA content.

### Clinical Trial

This is a randomized, prospective, interventional study (EudraCT: 2011-003541-17. August 2011). Male patients, aged between 25–40 years, who had one lower leg immobilized with a posterior ankle splint due to an ankle sprain (grade II), were identified when they attended the Emergency Department and given a study information sheet and invitation to take part in the study. If suitable and willing to take part, they underwent a full screening process and participants had their first MR scan. Patients were excluded if, they reported liver or gastrointestinal disease, infectious processes, untreated hypothyroidism, drug addiction, vitamin supplementation, eating or mental disorders, depression or anxiety, taking medication that decreases the concentration of lipids or taking antihypertensive drugs. We also excluded from the study athletes who exercised intensely and eligible patients who could not commit to attending for the second scan and blood extraction. Written informed consent was received from participants prior to inclusion in the study. The patients were allocated to the experimental groups by simple randomization using a computer program.

### Study Design

Subjects were invited for MRI and blood extractions on study days 0 and 15, with posterior ankle splint being applied on day 0. Seventy-three patients were assessed for eligibility in this study. Thirty-five patients agreed to participate in the clinical trial and 25 of these completed it. Fourteen patients had the right and 11 the left lower leg immobilized. Following randomization, 13 patients were treated with 300 mg·day^−1^ of allopurinol (orally), and 12 did not receive any treatment during all the immobilization period (See Supplementary Figure 4). The patients were recruited by 4 rehabilitation doctors from the “Hospital Universitario la Fe de Valencia”. Researchers responsible for data gathering were blinded for this study. This trial was registered on 22 November 2011. The patients’ recruitment started on 25 April 2012 and finished on 31 August 2015.

### MRI Protocols

All scans were acquired on two different General Electric 1.5 Tesla (T) MR Scanners (Signa HDxt and Optima MR360, General Electric, Milwaukee, USA) using a full-body coil. Both lower legs were imaged simultaneously. Patients lay in feet-first position, with the knee extended without loading.

The MRI sequence chosen for the soleus muscle segmentation was an ultra-fast Gradient-Echo (FSPGR). Acquisition parameters were as follows: TR/TE = 9/3 ms, 512 × 512 matrix, FA = 10°, Pixel Spacing = 0.6641–0.8203 mm, Slice Thickness = 5 mm, Spacing Between Slices = 2.5–5.0 mm. Images were acquired in axial planning, covering from the tibial tuberosity to the inferior tip of the lateral malleolus (used as reference landmarks to guarantee coverage homogeneity in the scans).

### Image Analysis

Image analysis and segmentation of the soleus muscle were performed with Mimics Research v17.0 (Materialise, Leuven, Belgium), under the supervision of a radiologist with more than 10 years of experience. A 3-step segmentation process was applied to the FSPGR MRI sequence. First, a gray level threshold was applied to the images to remove background elements and other tissues, such as bone or vessels. A region growing algorithm based on seed growth was then used to obtain a mask of the muscles of the leg under study. The final step consisted on a slice-per-slice manual refinement of the mask, removing other muscles, such as the gastrocnemius, and obtaining the final segmentation.

Imaging analysis was then performed on the resulting mask of the soleus muscle. The following imaging biomarkers were extracted:

- Volume [mL]
- Gray level mean intensity in the whole volume
- Gray level mean intensity (normalized) in the whole volume
- Gray level intensity standard deviation in the whole volume
- Gray level maximum intensity in the whole volume
- Gray level minimum intensity in the whole volume
- Maximum CSA [cm^2^]
- Gray level mean intensity in the CSA
- Gray level intensity standard deviation in the CSA
- Gray level maximum intensity in the CSA
- Gray level minimum intensity in the CSA

As an additional measurement, a 2D region of interest was identified in the gastrocnemius muscle in each study in order to calculate the gray level mean intensity and the standard deviation for normalization purposes.

### Laboratory measurements in humans

Blood was collected by venipuncture from the antecubital vein before and after the immobilization protocol. Serum and plasma were stored at −80 °C. Blood analyses were performed according to standard laboratory protocols at the central laboratory hospital (See Supplementary Table 1).

### Statistics

All results are expressed as mean ± SD. Statistical significance was considered with a p value of less than 0.05.

For the animal studies normality of distribution and homogeneity of the variance were checked with the Kolmogorov and Levene’s tests, respectively. One way ANOVA, and post hoc Bonferroni’s comparisons were used to evaluate statistical differences.

For the human image analysis, a non-parametric test of Mann-Withney was used to assess for significant differences. For the human blood parameters normality of distribution was checked with the Kolmogorov test, and homogeneity of variance was tested by Levene’s statistics. T-test was used to test for statistically significant differences between the groups.

## Electronic supplementary material

Supplementary Figures

## References

[1] Schiaffino S, Dyar KA, Ciciliot S, Blaauw B, Sandri M (2013). Mechanisms regulating skeletal muscle growth and atrophy. FEBS J.

[2] Bodine SC (2013). Disuse-induced muscle wasting. Int J Biochem Cell Biol.

[3] Reid MB, Judge AR, Bodine SC (2014). CrossTalk opposing view: The dominant mechanism causing disuse muscle atrophy is proteolysis. J Physiol.

[4] Gomes MD, Lecker SH, Jagoe RT, Navon A, Goldberg AL (2001). Atrogin-1, a muscle-specific F-box protein highly expressed during muscle atrophy. Proc Natl Acad Sci USA.

[5] Bodine SC (2001). Identification of ubiquitin ligases required for skeletal muscle atrophy. Science.

[6] Kedar V (2004). Muscle-specific RING finger 1 is a bona fide ubiquitin ligase that degrades cardiac troponin I. Proc Natl Acad Sci USA.

[7] Li HH (2004). Atrogin-1/muscle atrophy F-box inhibits calcineurin-dependent cardiac hypertrophy by participating in an SCF ubiquitin ligase complex. J Clin Invest.

[8] Lecker SH, Goldberg AL, Mitch WE (2006). Protein degradation by the ubiquitin-proteasome pathway in normal and disease states. J Am Soc Nephrol.

[9] Bodine SC (2001). Akt/mTOR pathway is a crucial regulator of skeletal muscle hypertrophy and can prevent muscle atrophy *in vivo*. Nat Cell Biol.

[10] Ogawa T (2006). Ubiquitin ligase gene expression in healthy volunteers with 20-day bedrest. Muscle Nerve.

[11] Nakao R (2009). Ubiquitin ligase Cbl-b is a negative regulator for insulin-like growth factor 1 signaling during muscle atrophy caused by unloading. Mol Cell Biol.

[12] Bonaldo P, Sandri M (2012). Cellular and molecular mechanisms of muscle atrophy. Dis Model Mech.

[13] Nikawa T (2004). Skeletal muscle gene expression in space-flown rats. Faseb J.

[14] Sandri M (2004). Foxo transcription factors induce the atrophy-related ubiquitin ligase atrogin-1 and cause skeletal muscle atrophy. Cell.

[15] Mammucari C (2007). FoxO3 controls autophagy in skeletal muscle *in vivo*. Cell Metab.

[16] Cai D (2004). IKKbeta/NF-kappaB activation causes severe muscle wasting in mice. Cell.

[17] Brocca L (2009). Is oxidative stress a cause or consequence of disuse muscle atrophy in mice? A proteomic approach in hindlimb-unloaded mice. Exp Physiol.

[18] Pellegrino MA (2011). Redox homeostasis, oxidative stress and disuse muscle atrophy. J Physiol.

[19] Davies KJA, Delsignore ME, Lin SW (1987). Protein damage and degradation by oxygen radicals. II. Modification of amino acids. J Biol Chem.

[20] Gomez-Cabrera MC (2010). Effect of xanthine oxidase-generated extracellular superoxide on skeletal muscle force generation. Am J Physiol Regul Integr Comp Physiol.

[21] Gomez-Cabrera MC (2005). Decreasing xanthine oxidase-mediated oxidative stress prevents useful cellular adaptations to exercise in rats. J Physiol.

[22] Gomez-Cabrera MC (2006). Oxidative stress in marathon runners: interest of antioxidant supplementation. Br J Nutr.

[23] Gomez-Cabrera MC, Pallardo FV, Sastre J, Vina J, Garcia-del-Moral L (2003). Allopurinol and markers of muscle damage among participants in the Tour de France. Jama.

[24] Derbre F (2012). Inhibition of Xanthine Oxidase by Allopurinol Prevents Skeletal Muscle Atrophy: Role of p38 MAPKinase and E3 Ubiquitin Ligases. PLoS One.

[25] Akima H (2001). Inactivity and muscle: effect of resistance training during bed rest on muscle size in the lower limb. Acta Physiol Scand.

[26] Akima H (2000). Leg-press resistance training during 20 days of 6 degrees head-down-tilt bed rest prevents muscle deconditioning. Eur J Appl Physiol.

[27] Akima H (2003). Resistance training during unweighting maintains muscle size and function in human calf. Med Sci Sports Exerc.

[28] Alkner BA, Tesch PA (2004). Knee extensor and plantar flexor muscle size and function following 90 days of bed rest with or without resistance exercise. Eur J Appl Physiol.

[29] Burks TN (2011). Losartan restores skeletal muscle remodeling and protects against disuse atrophy in sarcopenia. Sci Transl Med.

[30] Tsatsanis C, Androulidaki A, Venihaki M, Margioris AN (2006). Signalling networks regulating cyclooxygenase-2. Int J Biochem Cell Biol.

[31] Simmons DL, Botting RM, Hla T (2004). Cyclooxygenase isozymes: the biology of prostaglandin synthesis and inhibition. Pharmacol Rev.

[32] Glass DJ (2003). Molecular mechanisms modulating muscle mass. Trends Mol Med.

[33] Konishi M (2015). Febuxostat improves outcome in a rat model of cancer cachexia. J Cachexia Sarcopenia Muscle.

[34] Hanson AM, Harrison BC, Young MH, Stodieck LS, Ferguson VL (2013). Longitudinal characterization of functional, morphologic, and biochemical adaptations in mouse skeletal muscle with hindlimb suspension. Muscle Nerve.

[35] Baar K, Esser K (1999). Phosphorylation ofp70(S6k) correlates with increased skeletal muscle mass following resistance exercise. Am J Physiol.

[36] Lai KM (2004). Conditional activation of akt in adult skeletal muscle induces rapid hypertrophy. Mol Cell Biol.

[37] Tisdale MJ (2005). The ubiquitin-proteasome pathway as a therapeutic target for muscle wasting. J Support Oncol.

[38] Sanchis-Gomar F (2015). Allopurinol prevents cardiac and skeletal muscle damage in professional soccer players. Scand J Med Sci Sports.

[39] Beveridge LA, Ramage L, McMurdo ME, George J, Witham MD (2013). Allopurinol use is associated with greater functional gains in older rehabilitation patients. Age Ageing.

[40] Baldwin KM (1996). Musculoskeletal adaptations to weightlessness and development of effective countermeasures. Med Sci Sports Exerc.

[41] Schulze K, Gallagher P, Trappe S (2002). Resistance training preserves skeletal muscle function during unloading in humans. Med Sci Sports Exerc.

[42] Hotta N (2011). The effect of intense interval cycle-training on unloading-induced dysfunction and atrophy in the human calf muscle. J Physiol Anthropol.

[43] Matuszczak Y, Arbogast S, Reid MB (2004). Allopurinol mitigates muscle contractile dysfunction caused by hindlimb unloading in mice. Aviat Space Environ Med.

[44] Whidden MA (2009). Xanthine oxidase contributes to mechanical ventilation-induced diaphragmatic oxidative stress and contractile dysfunction. J Appl Physiol.

[45] Springer J (2012). Inhibition of xanthine oxidase reduces wasting and improves outcome in a rat model of cancer cachexia. Int J Cancer.

[46] Kelleher AR, Pereira SL, Jefferson LS, Kimball SR (2014). REDD2 expression in rat skeletal muscle correlates with nutrient-induced activation of mTORC1: responses to aging, immobilization, and remobilization. Am J Physiol Endocrinol Metab.

[47] Booth FW, Seider MJ (1979). Early change in skeletal muscle protein synthesis after limb immobilization of rats. J Appl Physiol Respir Environ Exerc Physiol.

[48] Langet H (2012). Compressed sensing dynamic reconstruction in rotational angiography. Med Image Comput Comput Assist Interv.

[49] Ferrando AA, Lane HW, Stuart CA, Davis-Street J, Wolfe RR (1996). Prolonged bed rest decreases skeletal muscle and whole body protein synthesis. Am J Physiol.

[50] Glover EI (2008). Immobilization induces anabolic resistance in human myofibrillar protein synthesis with low and high dose amino acid infusion. J Physiol.

[51] Murata M, Kosaka R, Kurihara K, Yamashita S, Tachibana H (2016). Delphinidin prevents disuse muscle atrophy and reduces stress-related gene expression. Biosci Biotechnol Biochem.

[52] Krogh-Madsen R (2010). A 2-wk reduction of ambulatory activity attenuates peripheral insulin sensitivity. J Appl Physiol (1985).

[53] Choi YJ (2014). Uric acid induces endothelial dysfunction by vascular insulin resistance associated with the impairment of nitric oxide synthesis. FASEB J.

[54] Brocca L (2017). FoxO-dependent atrogenes vary among catabolic conditions and play a key role in muscle atrophy induced by hindlimb suspension. J Physiol.

[55] Gomez-Cabrera MC, Vina J, Ji LL (2016). Role of Redox Signaling and Inflammation in Skeletal Muscle Adaptations toTraining. Antioxidants (Basel).

[56] Ohtsubo T, Rovira II, Starost MF, Liu C, Finkel T (2004). Xanthine oxidoreductase is an endogenous regulator of cyclooxygenase-2. Circ Res.

[57] Feng L, Xia Y, Garcia GE, Hwang D, Wilson CB (1995). Involvement of reactive oxygen intermediates in cyclooxygenase-2 expression induced by interleukin-1, tumor necrosis factor-alpha, and lipopolysaccharide. J Clin Invest.

[58] Chinery R (1998). Antioxidants reduce cyclooxygenase-2 expression, prostaglandin production, and proliferation in colorectal cancer cells. Cancer Res.

[59] Morey-Holton ER, Globus RK (2002). Hindlimb unloading rodent model: technical aspects. J Appl Physiol.

[60] Beckman JS, Parks DA, Pearson JD, Marshall PA, Freeman BA (1989). A sensitive fluorometric assay for measuring xanthine dehydrogenase and oxidase in tissues. Free Radic Biol Med.

[61] Ji LL, Gomez-Cabrera MC, Steinhafel N, Vina J (2004). Acute exercise activates nuclear factor (NF)-kappaB signaling pathway in rat skeletal muscle. Faseb J.

[62] Romagnoli M (2010). Xanthine oxidase-induced oxidative stress causes activation of NF-kappaB and inflammation in the liver of type I diabetic rats. Free Radic Biol Med.

[63] Dignam JD, Lebovitz RM, Roeder RG (1983). Accurate transcription initiation by RNA polymerase II in a soluble extract from isolated mammalian nuclei. Nucleic Acids Res.

