# Supplementary material for: Allopurinol partially prevents disuse muscle atrophy in mice and humans
Source: Sci Rep. 2018 Feb 23;8:3549. doi: 10.1038/s41598-018-21552-1 (PMC5824846; doi:10.1038/s41598-018-21552-1)
Supplement: Supplementary file 1 — Supplementary Figures [file 41598_2018_21552_MOESM1_ESM.docx]

***Research manuscript submitted to Scientific Reports***

**Title:** Allopurinol partially prevents disuse muscle atrophy in mice and humans

**Authors:** Beatriz Ferrando^1,2†^, Mari Carmen Gomez-Cabrera^2†^*, Andrea Salvador-Pascual^2^, Carlos Puchades^3^, Frederic Derbré^4^, Arlette Gratas-Delamarche^4^, Ludovic Laparre^4^, Gloria Olaso-Gonzalez^2^, Miguel Cerda^5^, Enrique Viosca^6^, Ana Alabajos^6^, Vicente Sebastiá^7^, Angel Alberich-Bayarri^8,9^, Fabio García-Castro^9^, Jose Viña^2^.

**Supplementary Table 1.** Biochemical parameters of the patients. Effect of the treatment with allopurinol.

|  | **Before lower**  **limb immobilization** | | **After lower**  **limb immobilization** | |
| --- | --- | --- | --- | --- |
|  | Control | Allopurinol | Control | Allopurinol |
| C-reactive protein (mg·L^-1^) | 3.90±4.44 | 3.93±5.72 | 1.69±1.11 | 1.90±1.63 |
| Leukocytes (10^3^·uL^-1^) | 8.56±2.71 | 8.41±2.94 | 6.91±0.96 | 7.36±1.60 |
| Neutrophils (10^3^·uL^-1^) | 5.40±2.27 | 5.92±2.97 | 4.00±0.70 | 4.67±1.60 |
| Lymphocytes (10^3^·uL^-1^) | 2.33±0.48 | 1.72±0.52 | 2.18±0.51 | 1.96±0.81 |
| Monocytes (10^3^·uL^-1^) | 0.66±0.21 | 0.56±0.27 | 0.58±0.13 | 0.47±0.16 |
| Eosinophils (10^3^·uL^-1^) | 0.14±0.12 | 0.18±0.13 | 0.15±0.08 | 0.23±0.15 |
| Basophils (10^3^·uL^-1^) | 0.02±0.04 | 0.00±0.00 | 0.01±0.02 | 0.01±0.01 |
| Hemoglobin (g·dL^-1^) | 15.35±0.72 | 14.69±1.00 | 15.47±0.64 | 14.97±0.85 |
| Hematrocrit (%) | 45.25±1.55 | 44.31±2.10 | 45.28±1.43 | 44.74±1.96 |
| Platelets (10^3^·uL^-1^) | 226.50±49.70 | 223.29±46.04 | 249.80±39.16 | 240.00±68.81 |
| Glucose (mg·dL^-1^) | 83.91±7.57 | 89.14±16.03 | 81.45±7.57 | 96.71±16.92 |
| Cholesterol (mg·dL^-1^) | 206.33±66.83 | 188.60±32.85 | 199.89±56.63 | 208.00±19.62 |
| Triglycerides (mg·dL^-1^) | 143.38±91.79 | 122.60±62.46 | 138.88±70.33 | 139.20±76.55 |
| Creatinine (mg·dL^-1^) | 0.92±0.11 | 0.92±0.15 | 0.92±0.10 | 0.89±0.08 |
| ALT/GPT (U·L^-1^) | 36.93±36.15 | 20.42±7.12 | 37.07±21.77 | 32.17±37.29 |
| AST/GOT (U·L^-1^) | 28.21±11.70 | 24.64±6.89 | 29.36±14.84 | 24.45±6.14 |
| Urea (mg·dL^-1^) | 38.22±7.08 | 28.00±10.86 | 35.32±6.76 | 31.50±6.61 |
| LDH (U·L^-1^) | 421.67±77.51 | 383.33±46.36 | 374.67±71.22 | 393.67±74.89 |
| Creatin Kinase (U·L^-1^) | 133.75±46.33 | 189.50±79.90 | 76.88±54.94 | 83.10±107.34 |

**Supplementary Figure 1.** Uncropped membranes for Figure 1D. Red square shows the samples used in this paper, samples outside it are from a different experiment. Only the full-length blots where the cropped gels were derived, have been included in supplementary information.

C

kDa


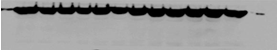


UA

U

kDa

223


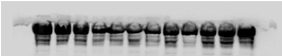


UA

C

U

MHC I

42

α actin

**Supplementary Figure 2.** Uncropped membranes for Figure 2A. Red square shows the samples used in this paper, samples outside it are from a different experiment. Only the full-length blots where the cropped gels were derived, have been included in supplementary information.


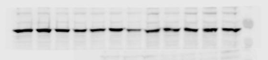


α actin

kDa

42

UA

C

U

25

kDa

MnSOD


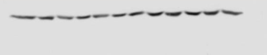


UA

C

U

38

P-p38


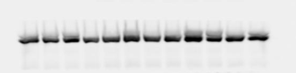


UA

C

U

42

α actin


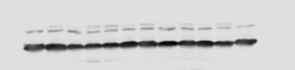


UA

C

U

38

p38

UA

C

U


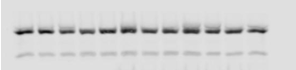


**Supplementary Figure 3**. Uncropped membranes for Figure 3A. Red square shows the samples used in this paper, samples outside it are from a different experiment. Only the full-length blots where the cropped gels were derived, have been included in supplementary information.

kDa


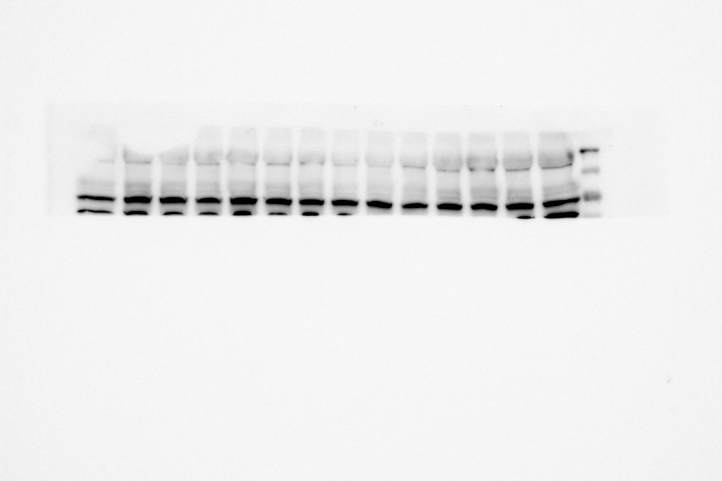

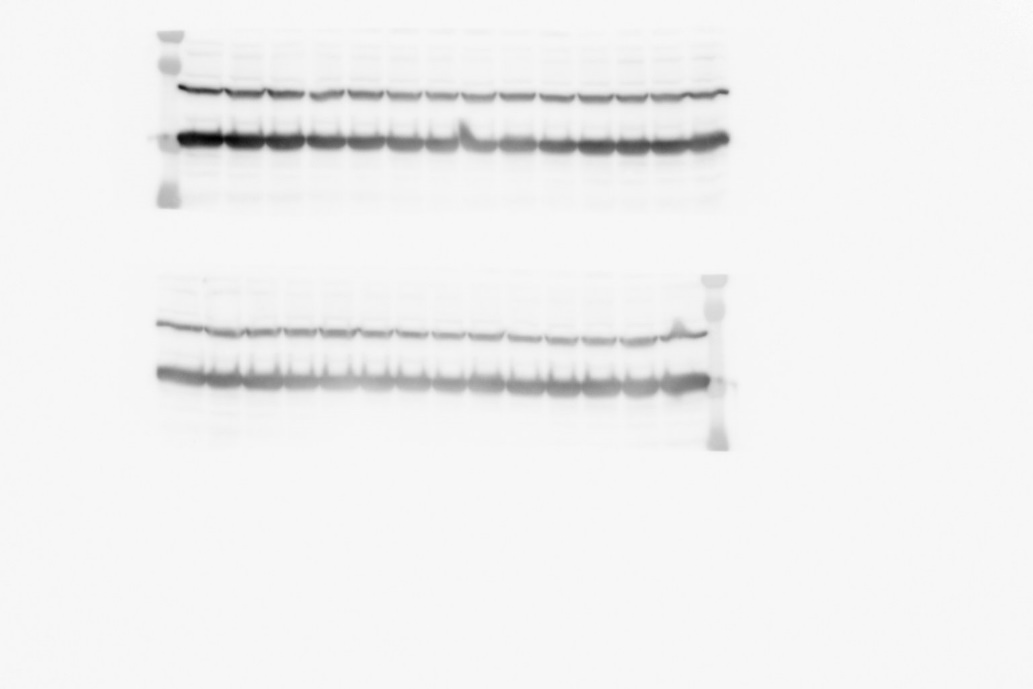


IRS-1

C

165

UA

U

37

C

UA

U

GAPDH

kDa

kDa


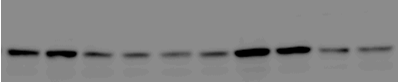


UA

C

U

p-Akt

70

UA

C

U

42


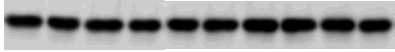


α Actin

Akt

70


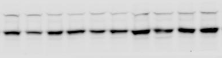


UA

C

U

p-FoxO3a


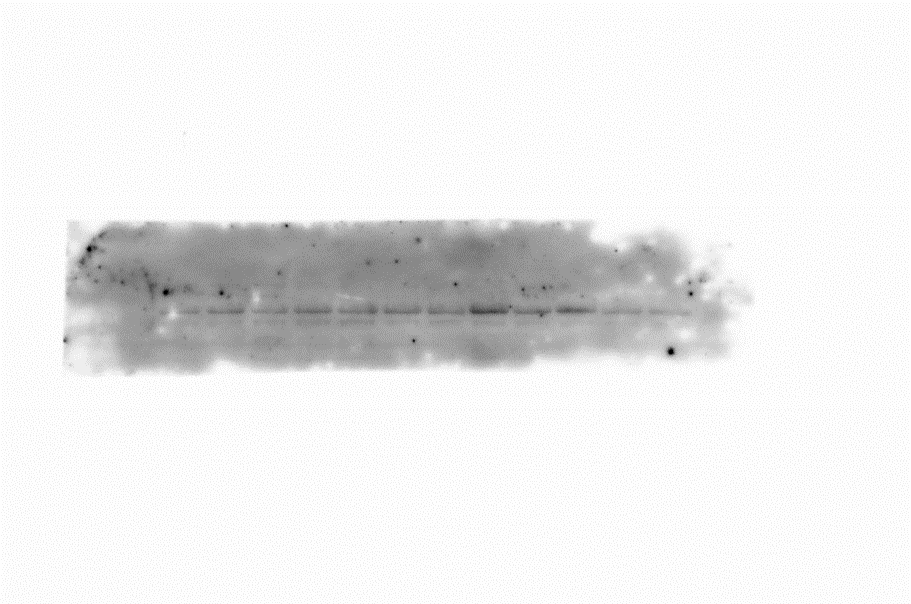


C

UA

UW

97

α Actin

C

UA

UW


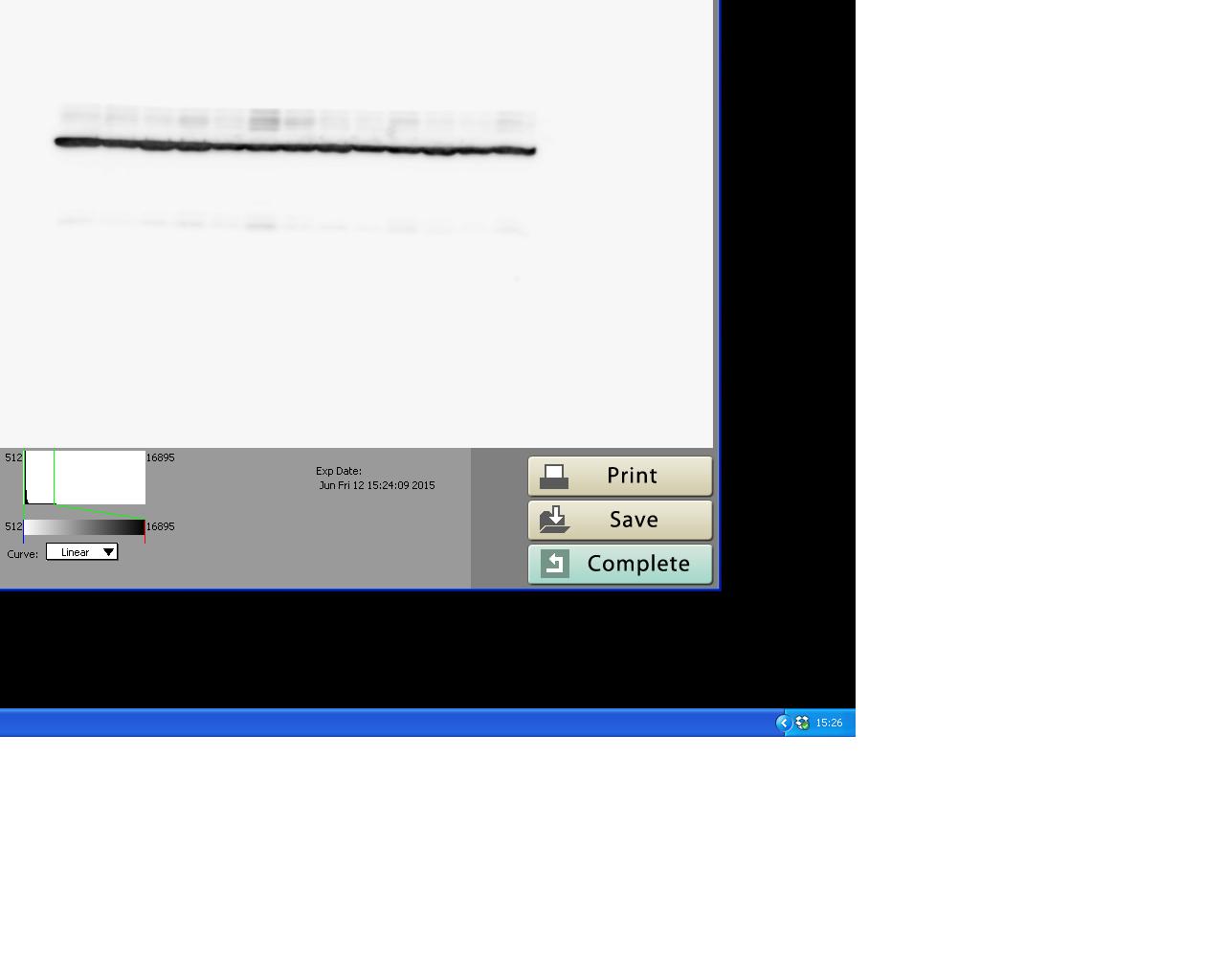


42

96


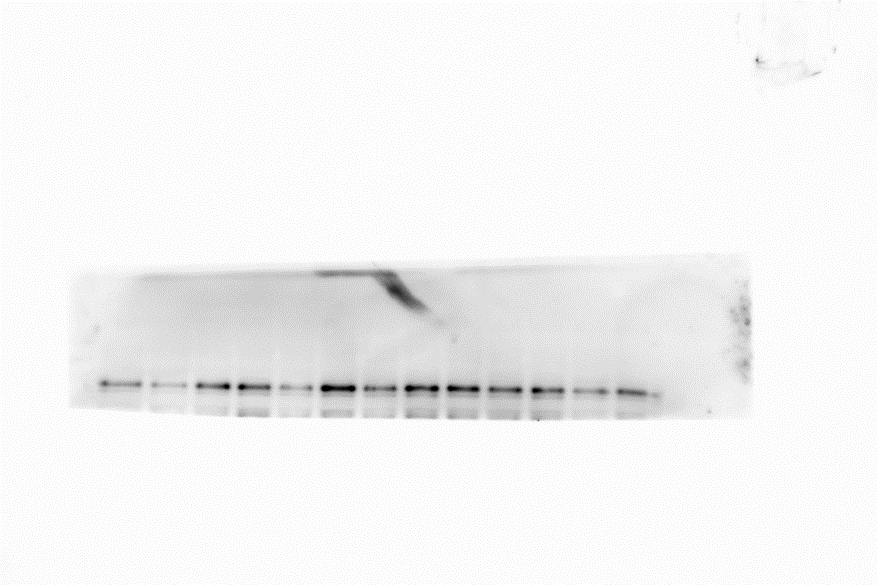


C

UA

UW

FoxO3a

ç

GAPDH

kDa

p62/SQSTM1

C


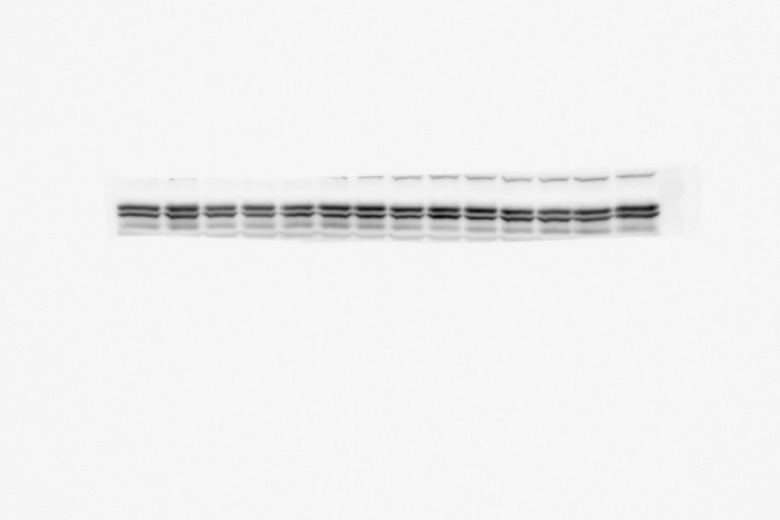


62

UA

U

kDa

37


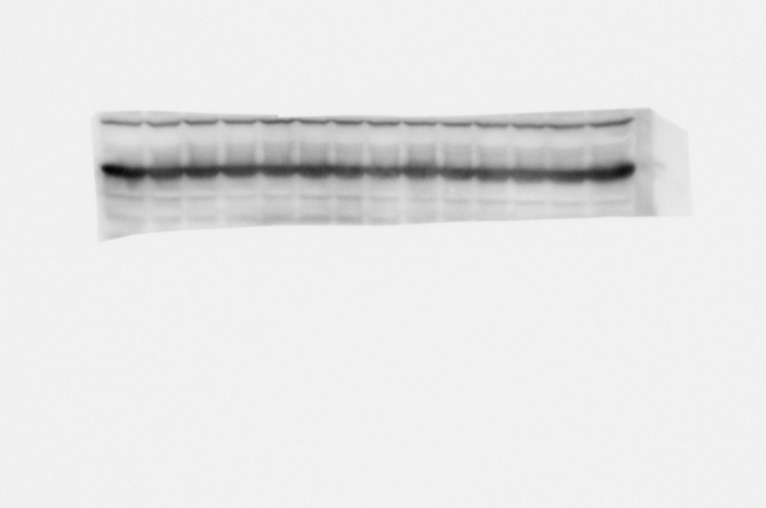


C

UA

U


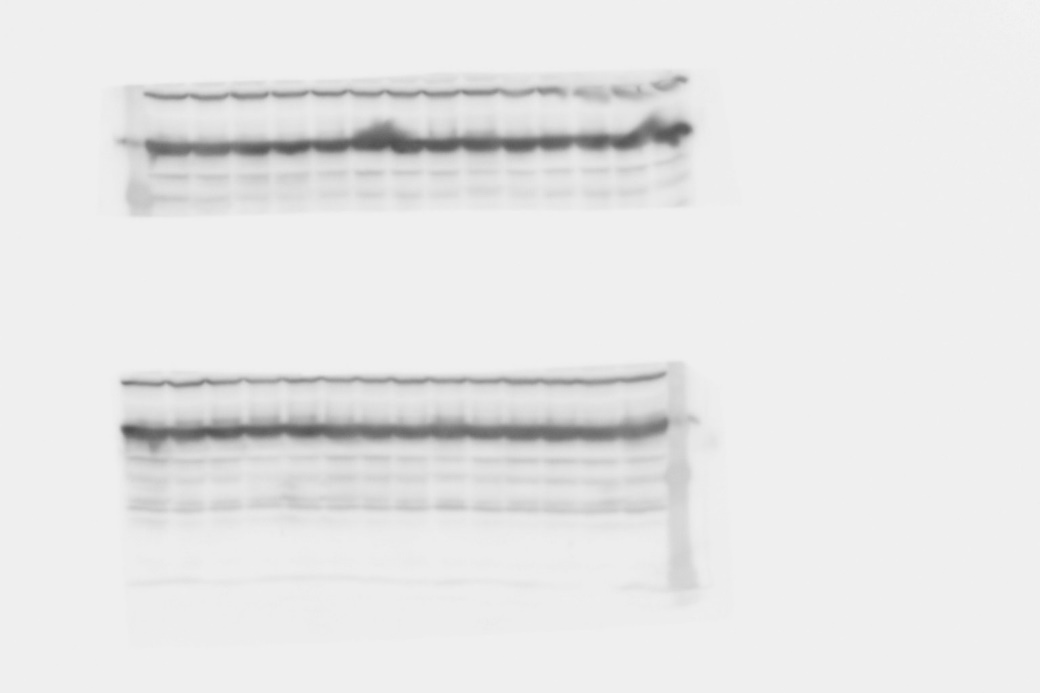

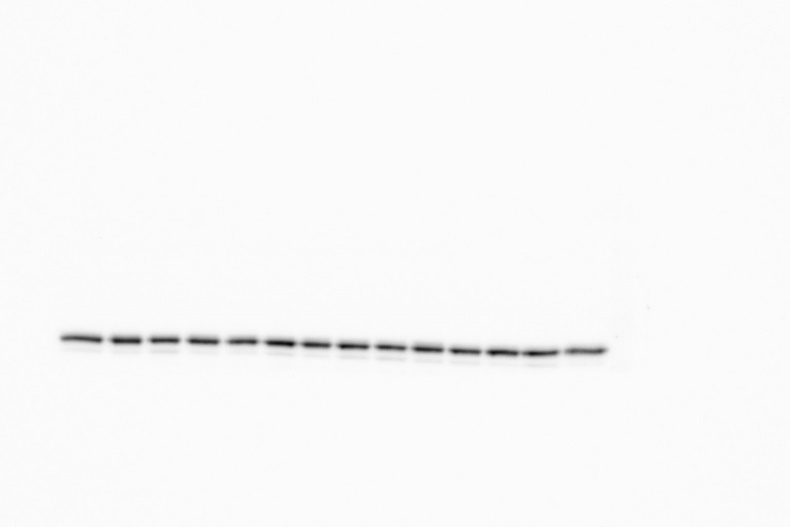


Beclin-1

C

60

UA

U

37

C

UA

U

GAPDH


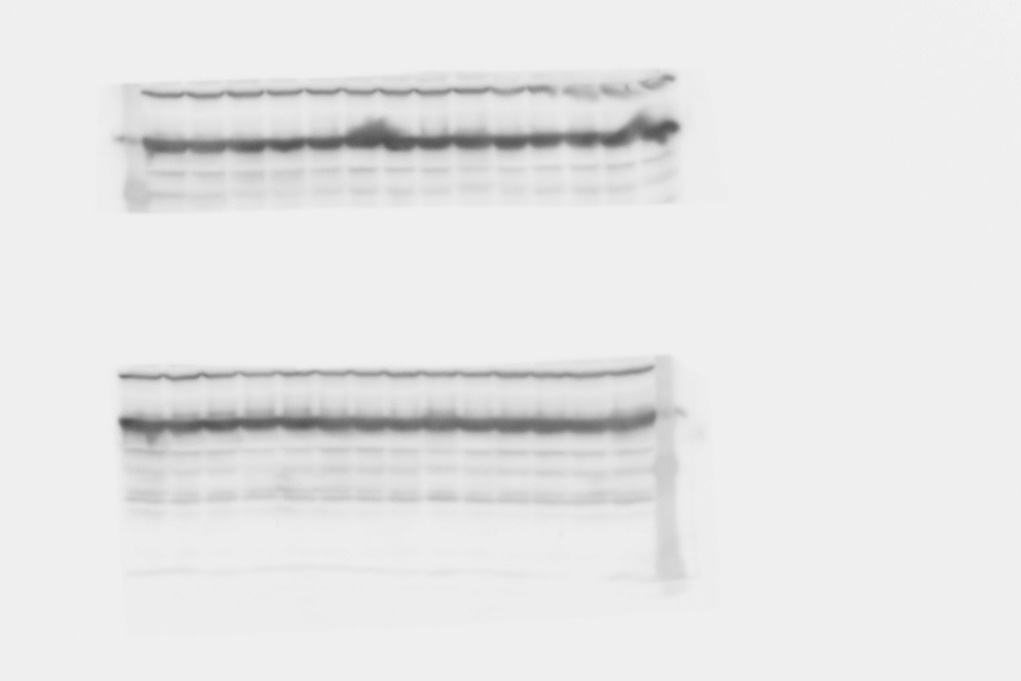


LC3

C

LC3 I: 16

LC3II: 14

UA

U

37

C

UA

U


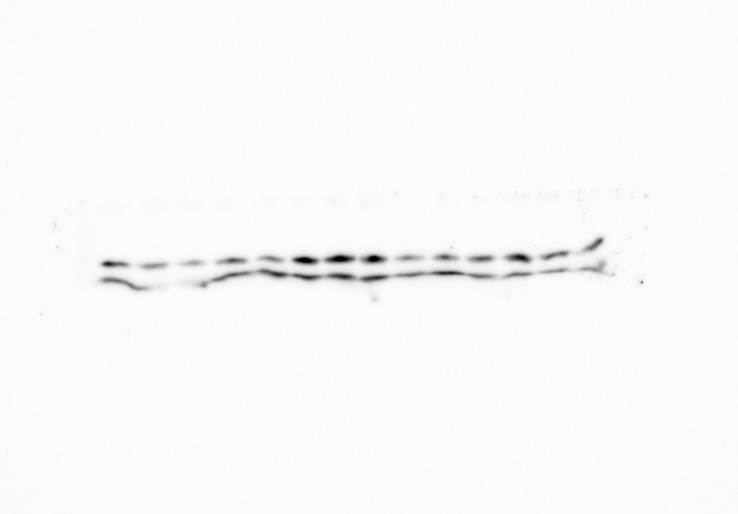


GAPDH

GAPDH

**Supplementary Figure 4.**  Subjects flow diagram from initial contact through study completion.

UA
